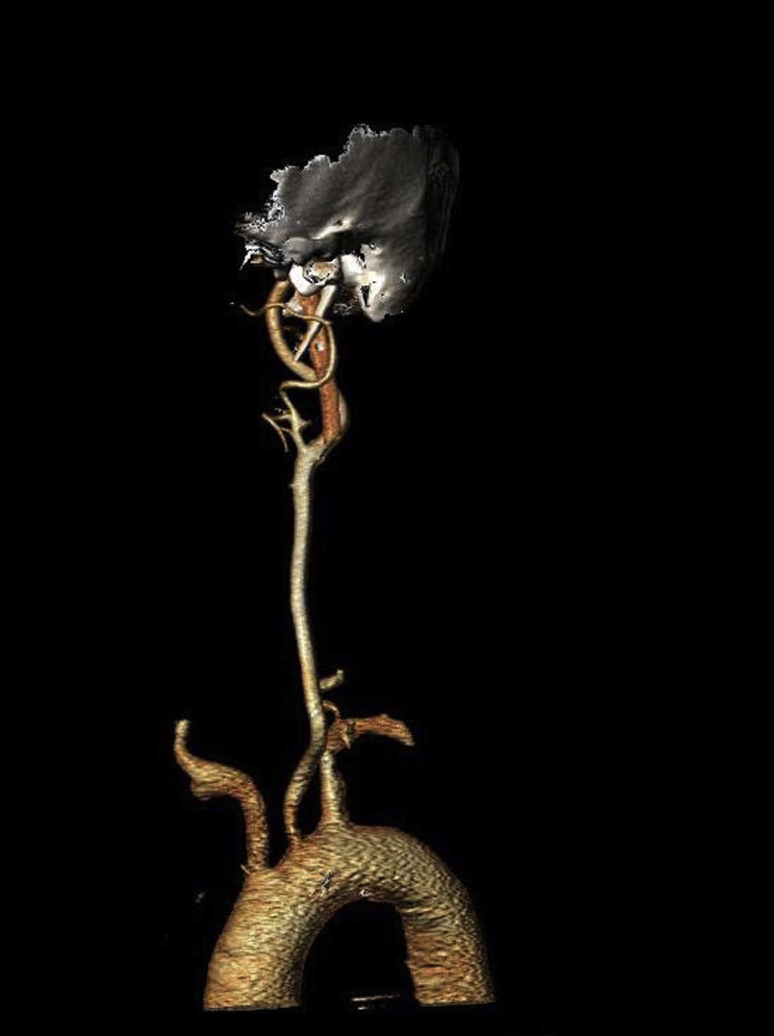# Refractory neck pain due to Eagle syndrome

**DOI:** 10.1016/j.jvscit.2021.01.006

**Published:** 2021-02-09

**Authors:** Sherif Sultan, Arielle Pierre, Yogesh Acharya, Niamh Hynes

**Affiliations:** a Western Vascular Institute, Department of Vascular and Endovascular Surgery, University Hospital Galway, National University of Ireland, Galway, Ireland; b Department of Vascular and Endovascular Surgery, Galway Clinic, Royal College of Surgeons in Ireland and National University of Ireland, Galway, Ireland

Eagle syndrome, named after Watt W. Eagle, who described the first cases in 1937, is an uncommon syndrome (4-8/10,000 persons) resulting from the styloid process's elongation or mineralization of the stylohyoid ligament.^1^ The typical symptoms are throat and neck pain radiating into the ear, which can be wrongly attributed to various facial neuralgias.

**Figure:**
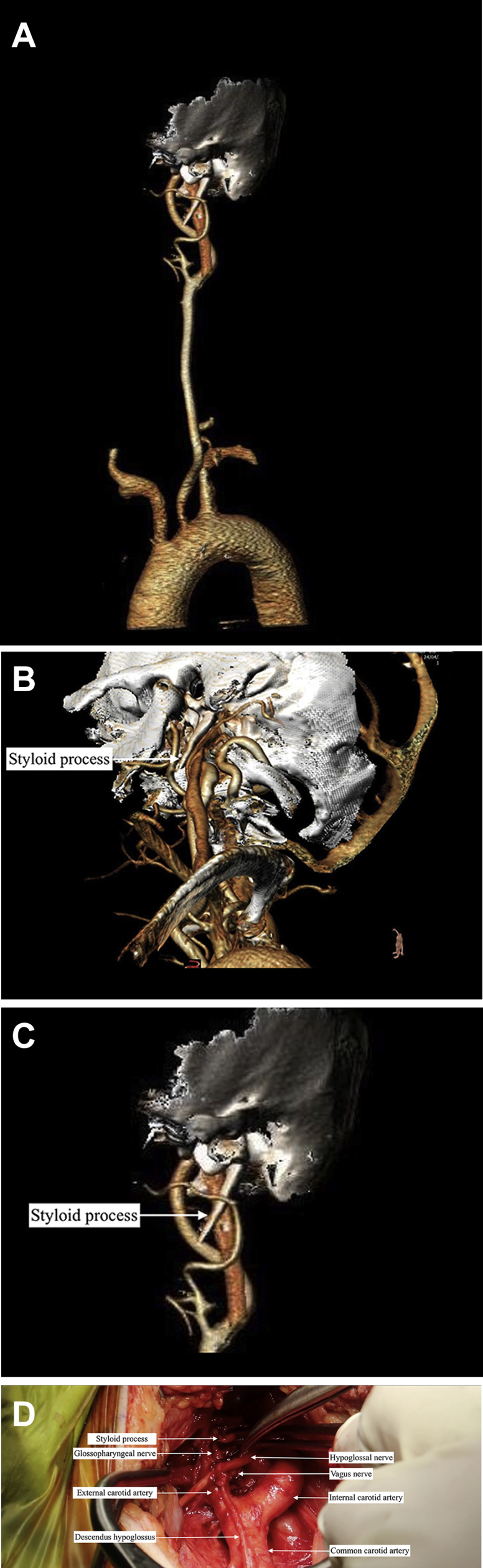

A 64-year-old woman had presented to the otolaryngology clinic with a slowly progressive intermittent dull pain in the upper neck of 10 years' duration. The pain was localized to the tonsillar fossa and radiated to the jaw, ear, neck, throat, and oropharynx. It was worsened by head movement and accompanied by dysphagia, a foreign body sensation, a sore throat, and voice changes. She had no history of trauma. The patient had undergone several consultations during the 10-year period, including otolaryngology, family practice, neurology, neurosurgery, psychiatry, and dentistry, with various interventions performed (ie, steroid injection in the temporomandibular joint, radiofrequency ablation, stellate ganglion block, lignocaine infusion, posterior auricular nerve block) without relief.

A computed tomography angiogram demonstrated a 4-cm-long styloid process at the level of the mid-internal carotid artery (*A* and *B*). Cervical neck incision and styloidectomy were planned. We exposed the common carotid artery and cranial nerves and retracted the posterior belly of the digastric to peel the tendinous attachment around the styloid process toward the mastoid posteriorly and mandible anteriorly for easy separation. The distal end of the styloid process was found to lie close to the hypoglossal, glossopharyngeal, and vagus nerves (*C*). A 2.5-cm excision of the styloid process was performed. She had complete resolution of the symptoms postoperatively. She had provided written informed consent for the report of her case details and images.

The styloid process normally measures 2.5 to 3 cm. However, when its length is >3 cm, the patient will become symptomatic owing to the subsequent pressure on the adjacent structures.^1^

Cervical neck surgery with styloidectomy is the definitive treatment. An alternative approach includes the intraoral transtonsillar route. However, this approach has been associated with high risks, including uncontrolled bleeding and cranial nerve injury because it limits visualization of the important structures.^2^

## Figures and Tables

**Cover image:**